# Prevalence and predictive testing preferences for breast cancer treatment side effects

**DOI:** 10.1007/s00520-025-09976-8

**Published:** 2025-11-20

**Authors:** Maura K. McCall, Demesvar Destin, Megan E. Miller, Fredrick R. Schumacher, Cheryl L. Thompson

**Affiliations:** 1 Frances Payne Bolton School of Nursing, Case Western Reserve University, Cleveland, OH USA; 2 Case Comprehensive Cancer Center, Cleveland, OH USA; 3 Department of Public Health Sciences, Penn State University College of Medicine and Penn State Cancer Institute, Hershey, PA USA; 4 University Hospitals Cleveland Medical Center, Cleveland, OH USA; 5 Department of Population and Quantitative Health Sciences, Case Western Reserve University School of Medicine, Cleveland, OH USA

**Keywords:** Breast cancer, Side effects, Quality of life, Biomarker testing preference

## Abstract

**Introduction:**

Women undergoing breast cancer (BC) treatment often face side effects that impact their quality of life and adherence. Biomarker tests to predict side effects could improve treatment options and adherence. This study assessed the prevalence of common side effects and women’s preferences for biomarker testing.

**Methods:**

Data from surveys of patients with BC at University Hospitals Cleveland Medical Center (UHCMC; *N* = 586) and Penn State Cancer Institute (PSCI; *N* = 562) were analyzed using descriptive statistics, chi-square, and Mann–Whitney *U*.

**Results:**

In the UHCMC and PSCI cohorts, 53.2% and 60.2% of the women prescribed endocrine therapy experienced joint and musculoskeletal pain (JMSP), respectively, with approximately one third discontinuing or changing treatment for BC. JMSP was associated with a higher mean body mass index (BMI). Over half of the women experienced hot flashes which were associated with younger age and Black race. Of the women receiving chemotherapy, nearly two thirds experienced neuropathy. Approximately one quarter of the respondents reported lymphedema, which as associated with a higher mean BMI, chemotherapy, and Black race. Quality of life was negatively impacted by side effects. Most women who experienced a side effect were more likely to prefer having a predictive test with the exception of women who experienced taxane-induced peripheral neuropathy.

**Conclusion:**

Side effects from BC treatment negatively affect quality of life and could be mitigated if their likelihood was known. Since the majority of BC patients view predictive biomarker testing favorably, future work focusing on treatment side effects is crucial to improve adherence, particularly to endocrine therapy.

**Supplementary Information:**

The online version contains supplementary material available at 10.1007/s00520-025-09976-8.

## Introduction

The 5-year overall survival rates have improved for women with breast cancer (BC) [1], resulting in more than 4 million women living with the diagnosis in the USA [2, 3]. However, although all races have seen improved 5-year survival over the last several decades, the mortality gap for Black women persists [3]. Women undergoing BC treatments often face a range of short- and long-term side effects that significantly impact their quality of life during and after treatment. These side effects vary based on the treatments received [2]. For instance, endocrine therapy (e.g. tamoxifen, anastrozole, letrozole, exemestane) can lead to numerous, varied side effects, including joint and musculoskeletal pain and hot flashes, two of the most studied side effects from BC therapies [6]. Joint and musculoskeletal pain (JMSP) ranges from 20 to 74% [4], and hot flashes occur in up to 78% of women taking endocrine therapy (ET) [5–7]. Black women with BC experience more pain and hot flashes than their White counterparts [8]. Among women without BC, Black and Hispanic women are generally twice as likely to experience hot flashes during menopause compared to White women, even after adjusting for medical history [9]. Additionally, BC, oncologic treatments (e.g., surgery, radiation), and associated comorbidities (e.g. overweight/obesity) may increase the risk of developing complications like peripheral neuropathy and lymphedema. Taxane-induced peripheral neuropathy (TIPN), a dose-limiting side effect of chemotherapy, is observed in 11–83% of patients, based on specific drug received [10]. TIPN prevalence differs across races, and risk factors for development include older age and body mass index (BMI) [10–13]. Lymphedema risk factors include, higher BMI, time, older age, Black race, Hispanic ethnicity, and receiving neoadjuvant chemotherapy [14, 15] though how to avoid lymphedema remains unclear [16]. In summary, these known cancer and treatment-related side effects and their presumptive risk factors have been studied to varying extent with mixed results. However, oncology and other fields have utilized genetic biomarkers such as *DPYD* variants to guide fluoropyrimidine dose adjustments [17, 18] and non-genetic biomarkers to predict side effects such as the association of serum bilirubin levels with irinotecan toxicity [19]. Thus, predictive biomarkers are an important tool to address side effect mitigation.

Utilizing precision health care to identify women at risk for side effects would enable clinicians to select the most appropriate treatments while avoiding suboptimal treatment adherence and provide early interventions for symptom management when side effects are likely. Developing biomarker tests to predict side effects may also assist with shared decision making among oncologists and patients as well as provide direction for targeted interventions to mitigate potential side effects. However, there is limited knowledge about patient preferences regarding biomarker testing and how such preferences may vary by self-reported race, as side effects themselves do. A systematic review spanning 20 years of research on attitudes and beliefs towards genetic testing indicated that Black participants are generally more wary of genetic testing compared with their White counterparts [20]. These concerns and beliefs were further demonstrated when targeted racial testing such as *APOL1* for Black kidney donors was implemented, enabling a potentially discriminatory decision [21]. These differences are, in part, thought to be associated with lower awareness levels and factual knowledge about genetic tests among racial and ethnic minorities [20]. Given the precision healthcare focus in BC and the known racial disparities in BC outcomes, information regarding racial preferences for potential biomarker risk factor testing, as well as barriers to its use, would be valuable to patients and clinicians, and potentially address disparities in treatment adherence and outcomes.

The main objectives of this study are to describe (1) women’s self-report of common BC treatment side effects (e.g., JMSP, hot flashes) and (2) the impact of side effects on quality of life, as well as (3) preferences for potential biomarker testing to help predict side effects and potential barriers to using biomarker testing among Black and White women.

## Methods

For this study, we used patient survey data, previously collected from a subgroup of patients diagnosed with BC who took part in a case–control study (2007–2013) at University Hospitals Cleveland Medical Center (UHCMC). A second independent sample and similar survey (2024) was conducted at the Penn State Cancer Institute (PSCI) among women currently diagnosed with and treated for BC, identified through electronic health record data. An IRB-approved, decedent-cleansed, de-identified email list was entered into REDCap®. For a comparison of study methods, see Table 1.

**Table 1:** Comparison of study methods

UHCMC cohort	PSCI cohort
Cross-sectional, one-time survey	Cross-sectional, one-time survey
Conducted in 2012–2013	Conducted in April–June, 2024
Recruited from parent study	Recruited for this study via an electronic de-identified list
*Eligibility criteria for parent study:*	*Eligibility criteria for EHR de-identified list:*
Diagnosed with female breast cancer within 3 years	Current diagnosis/treatment of female breast cancer
Receiving care at UHCMC	Receiving care at PSCI
No known *BRCA1/2* mutation	-
Parent study collected sociodemographic (age, self-reported race) and clinical data (cancer stage, receptor status, and treatments)	No specific sociodemographic or clinical data were collected
Pen and paper survey collected once with some missing data	Electronic survey collected once and most were complete responses were used for analysis, but participants could skip questions
Survey questions specified taxane-induced chemotherapy (TIPN)	Survey question asked about any chemotherapy-induced peripheral neuropathy (CIPN)

Note: See supplement for study flow charts

The 25-question pen-and-paper UHCMC survey was developed by parent study investigators. The survey was not validated. Participants completed the survey once to ascertain BC treatment side effects and patient preferences toward predictive biomarker testing. Participants were asked to recall their experience of cancer therapy side effects (‘yes’, ‘no’/’not sure’). We evaluated two symptoms—pain and hot flashes—for women prescribed ET (e.g., tamoxifen, anastrozole, letrozole, exemestane). A skip/fill pattern was used, e.g., if not on ET, the follow-up ET questions were skipped. We inquired if they received taxane-based chemotherapy, and, if so, did they experience taxane-induced peripheral neuropathy. We asked all participants if they experienced lymphedema. We asked whether side effects had an impact on their quality of life (‘not at all’, ‘a little’, ‘somewhat’, ‘a lot’), their level of satisfaction with explanation of side effects (‘very dissatisfied’, ‘dissatisfied’, ‘neutral’, ‘satisfied’, ‘very satisfied’). Finally, we elicited their views on testing biological predictors of risk for side effects. Most questions on the PSCI questionnaire mirrored the UHCMC survey questions with the exception of asking about *chemotherapy-induced* peripheral neuropathy (versus TIPN) and follow-up questions on the explanation of side effects (see Table 4).

### Protection of human subjects

UHCMC parent study protocol was approved by the UH Institutional Review Board (IRB) (#02–05–11). Informed consent was obtained from all individual participants included in the study. The PSCI protocol was approved by the Penn State University IRB (IRB#STUDY00024191). Consent was obtained/verified within REDCap® by completing the anonymous survey.

### Statistical analysis

Data were summarized with descriptive statistics, bivariate analyses, including stratification by race, were conducted using Chi-square, correlations, Mann–Whitney *U*, *t* test, and logistic regression with adjustments for age and BMI. Analyses were conducted using SPSS (IBM Corp. Released 2023. IBM SPSS Statistics for Macintosh, Version 29.0.2.0. Armonk, NY: IBM Corp.) for the UHCMC cohort data analysis. Some responses were dichotomized prior to analysis, e.g., ‘no’ and ‘not sure’. The PSCI cohort data were analyzed descriptively within REDCap®.

## Results

### UHCMC cohort

Women (*n* = 586) who completed the survey were on average 60.5 (SD = 10.6) years of age (Table 2) and most self-reported their race as White (91.3%). Most women had estrogen receptor-positive tumors (78%) and early-stage invasive (69.3%) BC and had *no* family history of BC (80%). Women reported receiving neoadjuvant chemotherapy (8.5%), adjuvant chemotherapy (30.4%), adjuvant ET (68.1%), adjuvant trastuzumab (8.5%), breast-conserving surgery (68.3%), and radiation therapy (56.7%). When stratifying characteristics by self-reported race, Black women had a higher body mass index (BMI) (*p* = 0.002). A greater proportion of Black women received adjuvant chemotherapy (*p* = 0.038) but a greater proportion of White women received taxane-based chemotherapy (*p* = 0.040), adjuvant ET (*p* = 0.003), and breast conserving surgery (*p* = 0.048). White women were also more likely to be diagnosed with estrogen receptor positive tumors (*p* < 0.001) (Table 2). There were no significant differences for the additional characteristics by self-reported race. Characteristics of women who reported being prescribed ET for treatment of their BC were similar to the total sample (see Supplemental Table 1 and Supplemental Fig. 1).

**Table 2:** Participant and clinical characteristics for UHCMC survey responders

Characteristic (*N* = 586)	Total	Black (*n* = 51)	White (*n* = 535)
Age, mean (SD)	60.5 (10.6)	60.9 (11.1)	60.4 (10.6)
Range	27–88	40–83	27–88
BMI mean (SD)*	28.1 (6.3)	**31.4 (7.5)**	**27.8 (6.1)**
Range	17–53	**18–48**	**17–53**
ER + tumor	457 (78)	**31 (60.8)**	**426 (79.6)**
Cancer stage			
Ductal carcinoma in situ	112 (19.1)	10 (19.6)	102 (19.1)
Stage 1	264 (45.1)	22 (43.1)	242 (45.2)
Stage 2	142 (24.2)	14 (27.5)	128 (23.9)
Stage 3	35 (6)	3 (5.9)	32 (6)
Stage 4	9 (1.5)	2 (3.9)	7 (1.3)
No family history of breast cancer	469 (80)	40 (78.4)	429 (80.2)
Neoadjuvant chemotherapy	50 (8.5)	7 (13.7)	43 (8)
Adjuvant chemotherapy	178 (30.4)	**24 (47.1)**	**154 (28.8)**
Adjuvant endocrine therapy	399 (68.1)	**31 (60.8)**	**368 (68.8)**
Adjuvant trastuzumab	50 (8.5)	5 (9.8)	45 (8.4)
Breast-conserving surgery			
None	3 (0.5)	**1 (2)**	**2 (0.4)**
Yes	400 (68.3)	**31 (60.8)**	**369 (69)**
Mastectomy	143 (24.4)	**19 (37.3)**	**124 (23.2)**
Radiation therapy	332 (56.7)	29 (56.9)	303 (56.6)

Note: N (%) reported for categorical variables, Bolding indicates p < 0.05; ER + = estrogen receptor positive; cancer, early-stage invasive = women diagnosed with stage 1 or 2 breast cancer. Treatments are not mutually exclusive. BMI N missing = 3 White N = 533, Black N = 50, SD standard deviation, BMI body mass index

**Fig. 1:**
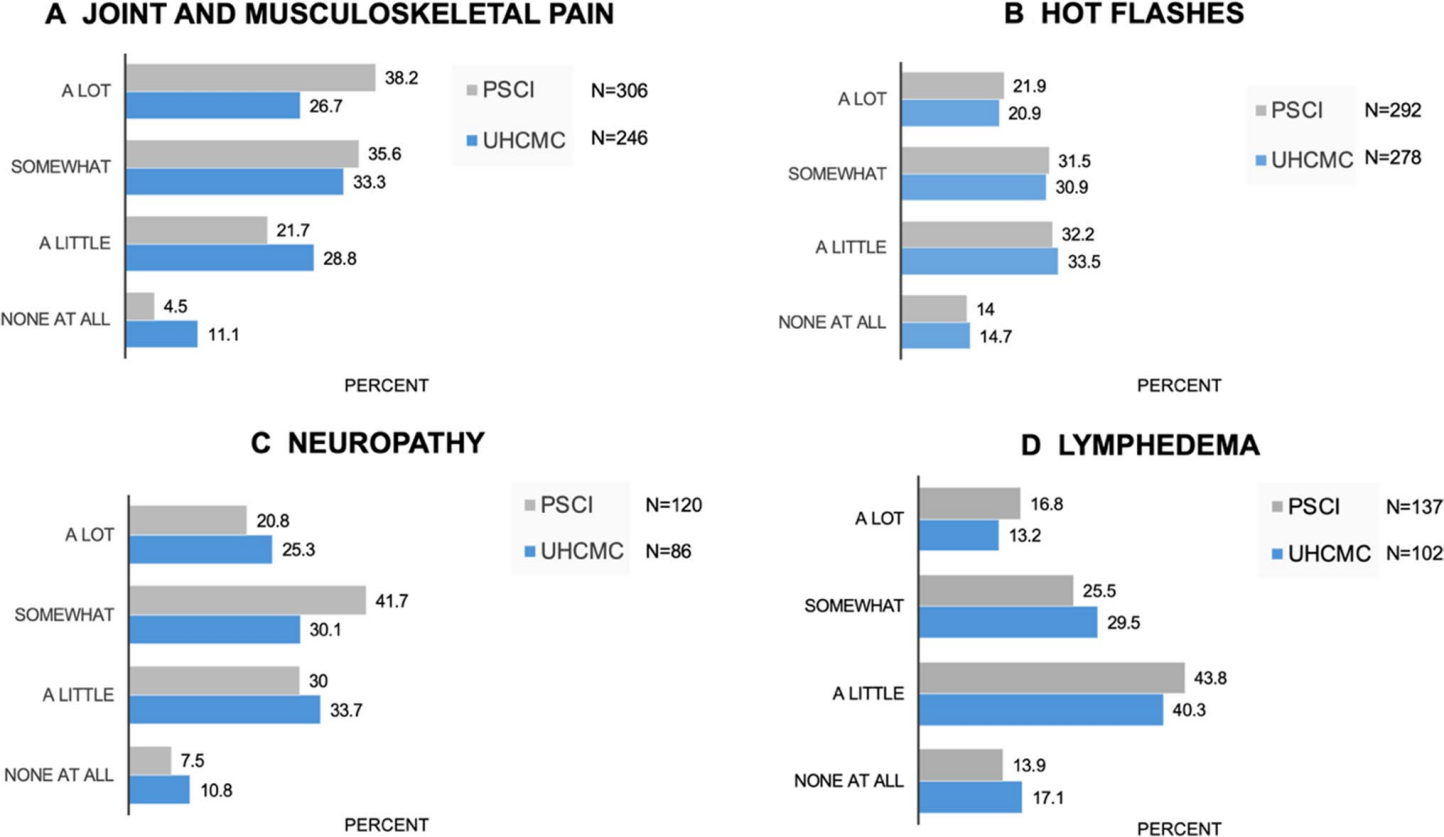
Extent to which each side effect affected quality of life in percent: Panel **A**. Joint and musculoskeletal pain (JMSP), Panel **B**. Hot flashes, Panel **C**. Neuropathy, Panel **D**. Lymphedema

Over half (53.2%) of 464 women prescribed ET experienced JMSP, of which 75 (30.4%) discontinued or changed treatment (Table 3). Discontinuation or medication changes due to JMSP did not differ by race and the JMSP/no pain groups did not differ by age or race. However, experiencing JMSP was associated with higher BMI (JMSP mean BMI = 28.9 SD = 6.4; no pain mean BMI = 27.0 SD = 5.8; *p* < 0.001). For the responses addressing quality of life for all side effects in both cohorts, see Fig. 1. The majority of women (82.1%) regardless of race indicated that their physician explained possible ET side effects, and most were ‘satisfied’ or ‘very satisfied’ with the explanation and the likelihood of their development (Supplemental Fig. 2).

**Table 3:** Survey results of treatment and symptom prevalence by cohort

Survey question	UHCMC cohort				PSCI
	Total *N* asked	Combined *N* (%)	Black *N* (%)	White *N* (%)	Total *N* (%)
Prescribed endocrine therapy	586	464 (79.2)	**29 (56.9)**	**435 (81.3)**	524 (93.2)
* If yes, to above:*	464				
Side effects were explained		381 (82.1)	22 (75.9)	359 (82.5)	-
Experienced joint/muscle pain		247 (53.2)	19 (65.5)	228 (52.4)	311 (60.2)
Stopped/changed treatment due to pain	247	75 (30.4)	4 (21.1)	71 (31.1)	98 (31.9)
Experienced hot flashes^a^		281 (60.6)	23 (79.3)	258 (59.3)	292 (56.4)
Stopped/changed treatment due to hot flashes	281	26 (9.3)	2 (8.7)	24 (9.3)	33 (11.3)
Received intravenous chemotherapy	586	256 (43.7)	**31 (60.8)**	**225 (42.1)**	207 (37.1)
* If yes, to above:*					
Taxane-based chemotherapy^b^	256	130 (50.8)	10 (32.3)	120 (53.3)	-
Side effects explained	130	125 (96.2)	10 (100)	115 (95.8)	-
Experienced TIPN/CIPN^c^	130	86 (66.2)	9 (90.0)	77 (64.2)	120 (58)
Stopped or changed treatment due to TIPN/CIPN	86	7 (8.1)	0 (0)	7 (9.1)	8 (6.7)
Experienced lymphedema (post-surgery)	586	130 (22.2)	**22 (43.1)**	**108 (20.2)**	138 (24.6)

Note: PSCI Total N = 562; UHCMC Total N 586 Black = 51/White = 535; with skip/fill questions, N changes based on response to a previous question. “Total N Asked” indicates the number of UHCMC participants expected to respond to the question. Bolding indicates p < 0.05. A dash—indicates that the question was not asked of that cohort, UHCMC University Hospitals of Cleveland Medical Center, PSCI Penn State Cancer Institute, TIPN taxane-induced peripheral neuropathy, CIPN chemotherapy-induced peripheral neuropathy

^a^Trend p = 0.06

^b^Trend p = 0.07

^c^Sample too small for comparison by race

More than half of women (60.6%) reported experiencing hot flashes, resulting in an ET change or discontinuation for 9.3% (Table 3). BMI was not associated with hot flashes experienced, but women who experienced hot flashes were significantly younger (57.4 ± 9.8 vs 65.9 ± 9.8; *p* < 0.001). Black women were greater than 5 times more likely to experience hot flashes compared with White women, adjusting for age and BMI (OR 5.326; 95% CI = 1.796, 15.792; *p* = 0.003, see Table 4). There was no difference by race for ET change or discontinuation.

**Table 4:** Logistic regression results for side effects by self-reported race, unadjusted, and adjusted for age and BMI

**Joint and musculoskeletal pain**						
		**Unadjusted**			**Adjusted**	
*Predictor*	*Odds ratio*	*95% CI*	*p*	*Odds ratio*	*95% CI*	*p*
Race	1.717	0.780, 3.777	0.179	1.446	0.638, 3.275	0.377
Age	0.998	0.971, 1.005	0.160	0.984	0.967, 1.002	0.076
BMI	1.052	1.019, 1.085	0.002	1.052	1.019, 1.086	0.002
**Hot flashes**						
		**Unadjusted**			**Adjusted**	
* Predictor*	*Odds ratio*	*95% CI*	*p*	*Odds ratio*	*95% CI*	*p*
Race	3.084	1.151, 8.269	0.025	5.326	1.796, 15.792	0.003
Age	0.917	0.898, 0.937	< 0.001	0.912	0.892, 0.933	< 0.001
BMI	0.988	0.959, 1.018	0.430	0.986	0.953, 1.020	0.420
**Taxane-induced peripheral neuropathy**						
		**Unadjusted**			**Adjusted**	
*Predictor*	*Odds ratio*	*95% CI*	*p*	*Odds ratio*	*95% CI*	*p*
Race	5.026	0.616, 41.019	0.132	4.532	0.544, 37.775	0.163
Age	0.999	0.961, 1.038	0.953	0.994	0.956, 1.035	0.780
BMI	1.071	1.007, 1.140	0.030	1.069	1.004, 1.139	0.038
**Lymphedema**						
		**Unadjusted**			**Adjusted**	
*Predictor*	*Odds ratio*	*95% CI*	*p*	*Odds ratio*	*95% CI*	*p*
Race	3.222	1.766, 5.877	< 0.001	2.792	1.495, 5.214	0.001
Age	0.985	0.967, 1.004	0.119	0.984	0.965, 1.003	0.098
BMI	1.043	1.012, 1.074	0.006	1.036	1.005, 1.068	0.023

Note: For the self-reported race variable (White, Black), White is the reference, CI confidence interval, BMI body mass index

More than one third (n = 256) of women in the overall sample reported receiving chemotherapy, half (*n* = 130) of whom received taxane-based chemotherapy. Nearly all (96.2%) reported that taxane side effects were explained. Most women (85.6%) were ‘very satisfied’ or ‘satisfied’ with the explanation of taxane-based chemotherapy side effects, and slightly fewer (80.8%) felt the same about the explanation for likelihood of developing side effects (Supplemental Fig. 3). Of those receiving taxane-based chemotherapy, 86 (66.2%) experienced taxane-based peripheral neuropathy (TIPN), and seven White women and no Black women stopped or changed their treatment due to TIPN. TIPN did not differ by age but differed by BMI with TIPN being associated with a higher BMI (TIPN mean BMI = 29.6, no TIPN mean BMI = 26.9; *p* = 0.014). TIPN by race was not significant in regression (Table 4), but the sample was small.

Lymphedema was reported by 22.2% (*n* = 130) of the overall sample. Age was not associated with lymphedema, but higher BMIs were associated significantly with lymphedema (lymphedema mean BMI = 29.5, no lymphedema mean BMI = 27.7; *p* = 0.005). Radiation (yes/no) and lymphedema were not related in this sample, but receiving any chemotherapy was significantly associated (*p* < 0.001). Black women had greater odds of developing lymphedema than White women adjusting for age and BMI (OR 2.792, 95% CI = 1.495, 5.214; *p* = 0.001).

### PSCI cohort

Of the 1872 surveys sent, 649 (29.5%) submissions were received and 562 were complete responses. Of the women who reported being prescribed ET (*n* = 524), 311 (60%) experienced JMSP and 98 (32%) stopped or changed their treatment as a result. Of those who experienced JMSP, 234 (76.5%) expressed interest in a blood test that can predict JMSP from ET. A total of 292 (56%) women experienced hot flashes from their ET, and 33 (11%) of them stopped or changed their treatment as a result.

Of the 207 women who reported receiving intravenous (IV) chemotherapy, 120 (59%) experienced neuropathy and 8 (7%) of them stopped or changed their treatment. Most reported being ‘satisfied’ or ‘very satisfied’ with how well the likelihood of developing side effects was explained to them. (see Supplemental Fig. 4 for satisfaction with the explanation). Twenty-five percent of all respondents (*n* = 138) reported experiencing lymphedema (Table 3).

### UHCMC cohort preferences for biomarker testing

Most women prescribed ET (71.3%) would prefer to have a blood test to predict JMSP, and there was no difference by race. Of the 247 women experiencing JMSP, 76.5% were in favor of a test to predict JMSP. Women who experienced JMSP were more likely to be interested in a test to predict JMSP (*p* = 0.012). Of women prescribed ET, two thirds (65.5%) would prefer to know results of a test predicting risk of developing hot flashes, and responses did not differ by race. Of the women who experienced hot flashes, 75.7% would prefer a blood test to predict hot flashes (*p* = 0.044). There was no difference by race. There was no difference in interest in a blood test to predict neuropathy due to chemotherapy based on experiencing TIPN or by race. Our survey did not include a question on predictive testing for lymphedema.

When asked generally about interest in a non-genetic blood test to predict side effects from cancer treatments, overall, more than half were interested (60.4%) but responses trended (*p* = 0.06) toward a difference by race, with Black women being less interested than White women. Of note, 19 (38.8%) Black women and 141 (26.7%) White women responded ‘not sure’. There were no differences by race on whether use of genetic testing to predict side effects of cancer treatments would change their interest in the test (Table 5). For those with greater concerns about such a test, ‘personal privacy’, ‘impact for others sharing some of my genes’, and ‘insurance company finding out’ were not statistically different by race (Table 5).

**Table 5:** Survey results of biomarker test preference by cohort

Survey question	UHCMC				PSCI
	Total *N* Asked	Combined *N* (%)	Black *N* (%)	White *N* (%)	Total *N* (%)
**Prescribed endocrine therapy**	586	464 (79.2)	**29 (56.9)**	**435 (81.3)**	524 (93.2)
*If yes to above (PSCI yes to the side effect):*					
For a blood test to predict pain, yes	464	331 (71.3)	**17 (58.6)**	**314 (72.1)**	235 (75.6)
For a blood test to predict hot flashes, yes	464	304 (65.5)	19 (65.5)	285 (65.5)	183 (62.7)
**Received chemotherapy **	586	256 (43.7)	**31 (60.7)**	**225 (51.7)**	207 (36.8)
UHCMC received taxane-based chemotherapy	256	130 (50.7)	**10 (33.3)**	**120 (53.3)**	-
*If yes to above, would you be interested in a blood test to predict TIPN (peripheral neuropathy): *	130				
Yes, absolutely (UHCMC) Yes (PSCI)		72 (55.4)	7 (70)	65 (54.2)	116 (56)
Yes, but only if my risk was much higher		36 (27.7)	1 (10)	35 (29.2)	53 (25.6)
**PSCI only: If there was a test available for predicting side effects of cancer treatment using information from your medical records would you have been interested in having it?**					420 (74.7)
**If there was a test available for predicting side effects of cancer treatment that does not look at genes, would you have been interested in having it?**					-
Yes^a^	586	349 (59.6)	**22 (43.1)**	**327 (61.1)**	-
**If a test (using DNA) to predict side effects existed, would that change interest in the test:**					
Yes, think more highly of it	586	158 (27)	7 (13.7)	151 (28.2)	190 (33.8)
No		218 (37.2)	22 (43.1)	196 (36.6)	207 (36.8)
I'm not sure		153 (26.1)	16 (31.4)	137 (25.6)	125 (22.2)
Yes, would have greater concerns		48 (8.2)	4 (7.8)	44 (8.2)	23 (4.1)
If yes to greater concerns, what reasons for those concerns:*	48				
a) personal privacy		20 (41.7)	2 (50)	18 (40.1)	13 (59.1)
b) impact for others that share some of my genes		33 (68.8)	3 (75)	30 (68.2)	11 (50)
c) my insurance company finding out		20 (41.7)	1 (25)	19 (43.2)	2 (9.1)

Note: PSCI Total N = 562 for bolded questions; UHCMC Total N 586 Black = 51/White = 535; with skip/fill questions, N changes based on response to a previous question. “Total N Asked” indicates the number of UHCMC participants expected to respond to the question. Bolding result indicates p < 0.05. A dash—indicates that the question was not asked of that cohort. TIPN taxane-induced peripheral neuropathy, DNA deoxyribonucleic acid and refers to using a ‘genetic’ test.

^a^Trend p = 0.06.

*Answers a–c are not mutually exclusive and only women who answered “yes, would have greater concerns” to “if a test (using DNA) to predict side effects existed, would that change your interest in the test” responded to the greater concerns a–c.

### PSCI cohort preferences for biomarker testing

Of the women who experienced JMSP (*n* = 311), 75.6% (*n* = 235) were interested in a blood test that could predict the likelihood of developing JMSP from endocrine treatment. Of those who experienced hot flashes (*n* = 291), 63% (*n* = 183) were interested in a similar test that could predict the likelihood of developing hot flashes from ET. Of those receiving IV chemotherapy, 81.6% (*n* = 169) were interested in a blood test that could predict the likelihood of developing significant neuropathy as a side effect..

For a PSCI additional question, 420 women (74.7%) reported that, thinking back to when they were deciding on treatment, they would have been interested in a test that could predict side effects from cancer treatment using only information from their medical records. Asked whether they would be interested in a similar test that also used DNA data, 190 women (33.8%) answered ‘Yes, I would think more highly of it’ while 23 (4%) answered ‘Yes, I would have greater concerns about this test’. Of the latter group, 13 indicated ‘privacy’ as a concern while 11 were more concerned with ‘impact for others sharing some of my genes’. Only two were concerned about their ‘insurance company finding out’ (Table 5).

## Discussion

We reported on results from two cohorts of women with BC who responded to a survey on common treatment-related side effects and predictive testing preferences. This study is distinctive in its use of two independent cohorts to examine (1) the prevalence of several common treatment-related side effects experienced by women with BC; (2) the impact of these side effects on quality of life; (3) women’s preferences for biomarker-based predictive testing for treatment-related side effects; and (4) comparing differences by self-reported race.

In our UHCMC cohort, our analysis revealed that approximately half of women who were prescribed ET experienced JMSP. We found an increase in this prevalence in our second survey, with 60% of women reporting JMSP from ET. Both figures align with other studies [4, 22]. The observed difference in JMSP prevalence between cohorts may be impacted by recall bias. UHCMC cohort participants, who may have been up to 3 years into a 5-year ET regimen, might not have accurately recalled their JMSP experiences, particularly if the side effect patterns changed temporally. In contrast, the PSCI cohort, who were currently undergoing treatment, likely provided a more accurate recollection of JMSP. This aligns with a small study of recall bias in osteoarthritis pain that reported that retrospective pain scores at 1- and 2-month timepoints were higher than those obtained from ecological momentary assessments [23]. While no statistically significant age or race-based difference was found between the JMSP and no pain groups, we found that a higher BMI was associated with JMSP, which reflects the current literature [24–26]. Although we did find a statistically significant difference in BMIs for Black versus White women, it’s important to note that there is an ongoing debate on the utility of BMI in statistical analyses and associations, as recommended BMI cutoffs for obesity and overweight classifications differ among races and ethnicities [27].

We also found the prevalence of hot flashes across both cohorts at 61% and 56% was concordant with values reported in the literature of about 22–83% [28]. Our finding that younger age was associated with hot flashes [7] and Black women were greater than five times more likely to have hot flashes after adjusting for age and BMI are consistent with other studies [8, 29].

We could not sufficiently test TIPN by race in our sample because of the small sample size, but racial disparities for TIPN are acknowledged and linked to patient and clinical factors as well as genetic variations [30]. Unfortunately, there has not been sufficient evidence to support pharmacogenomic guidelines for taxane administration [31]. Age was not associated, but a higher BMI was associated with TIPN. This association of TIPN with BMI may be linked to the use of body surface area to calculate chemotherapy dosage [32, 33]. Prevalence of CIPN was lower in our second survey, with 58% of women reporting neuropathy compared to 66% in the original survey. This decrease is encouraging though we do not know the reason for the decrease.

Importantly, we found that Black women in our study were about two and a half times more likely to develop lymphedema than White women after adjusting for age and BMI. In the general population, Black women are 3.5 times more likely to develop lymphedema than White women [14], which is in concordance with our unadjusted results. We found that a higher BMI was strongly associated with lymphedema, which aligns with findings from previous studies [34]. In this study, receiving any chemotherapy was significantly associated with lymphedema, but radiation was not. However, one study showed that lymphedema is multifactorial, including variables for which we did not have sufficient detail to test [35]. Due to the limitations of this descriptive study including available data and feasibility of multivariable analyses, further studies regarding racial differences and lymphedema risk factors are warranted.

It was not surprising to find that women who had JMSP were more likely to prefer a predictive biomarker test or that most women would like a test to predict hot flashes. However, when it came to TIPN, women were not as interested in a predictive test. One of the differences in the cohorts is when they were collected, with clinical biomarkers being more known than a decade ago. This is a possible explanation for differences in preferences for a predictive test result. The UHCMC data were collected years ago, and the patient familiarity with biomarker testing and genetics may have differed from the recent PSCI cohort collected in the past year. Additionally, the route of administration may have influenced attitudes towards the prescription. The patient may perceive that ET taken orally is not as ‘necessary’ or that ET can more easily be changed to another medication if one is causing bothersome side effects. In contrast, intravenous chemotherapy, may be considered a more ‘important’ or ‘necessary’ treatment, with oncologic benefit outweighing side effect risk. As one woman noted in the comments, “*I feel that side effect issues were a minor issue when opting for treatment. My concerns were survival percentages…”.*

Whereas previous studies have explored general beliefs and attitudes towards any biomarker testing between self-reported White and racial and ethnic minority populations, we investigated individual preferences for a biomarker test that can predict side effects specifically from BC treatments. Future studies should include questions about patients’ attitudes toward the risk/benefit ratio of BC treatments and their importance to cancer outcomes as well as quality of life.

Our results were mixed with respect to racial differences in preference for biomarker testing. Interest in this potential biomarker test, with or without a genetic component, was generally acceptable in more than half of our cohorts. Similar acceptability was reported in a previous survey of Black and White women with BC [36]. Approximately 8% had greater concerns for using DNA in a predictive test for the UHCMC cohort, but this decreased to 4% in the PSCI cohort, which was conducted more recently and may reflect changes in perceptions over time. In all, few women indicated that personal privacy, impact for others, and discovery of results by insurance company were major reasons for the concern for the test. While this may reflect improvement in patient understanding of biomarker testing, researchers and clinicians should communicate potential benefits and harms with their patients and elucidate patients’ preferences, particularly in the context of historical and structural factors that lead to mistrust in research and healthcare. Facilitators for potential use of biomarkers in future work include personal control, individual and community benefit, and transparent communication [21].

Several factors limit the interpretation of our results. Our sample consisted predominantly of White women in the first survey, and no ethnicity data was collected in the second survey. This reduces the overall generalizability of the findings. While some descriptive comparisons were made based on similarity of questions asked, no statistical interpretation could be made between the cohorts. The method of data collection pen and paper versus electronic online entry was different between the cohorts and may have influenced responses and the quantity of missing responses, particularly for the UHCMC cohort. Finally, as our data were almost entirely self-reported and retrospective, there is a possibility of inaccurate recall from respondents, particularly in the UHCMC cohort. Prospective side effect and biomarker studies may improve design by including patient perspectives.

Nevertheless, our study highlights that despite the many advances in BC care over the past decade, treatment-related side effects continue to affect women with BC and their quality of life. However, most women with BC are amenable to using a proactive precision health care approach to predict their likelihood of developing treatment-related side effects. Future research into the implications of social determinants of health (SDoH) should be assessed to gain a better understanding of the role played by variables such as education, income, and environment on the prevalence of those side effects among patients with BC, as well as preferences for biomarker and/or genetic testing. Despite interest in testing, clinically implemented biomarkers to predict side effects of BC treatment have been elusive. For example, research into use of pharmacogenomics [37] to clarify how genetic variants may affect drug metabolism and subsequent side effects from BC treatments such as aromatase inhibitors have not been definitive [38]. While there are Clinical Pharmacogenetics Implementation Consortium (CPIC) recommendations for tamoxifen and *CYP2D6* variants [39], there are no CPIC recommendations for aromatase inhibitors at this time. A predictive biomarker test has potential to help improve BC treatment adherence and provide an opportunity to change or adjust a treatment and/or mitigate side effects. Since the AIs have been shown to have similar efficacy, a proactive approach to side effect surveillance, coupled with targeted, early implementation of side effect management strategies may enhance symptom control, improve quality of life, and empower women at high risk for treatment-related side effects to actively participate in their care. Although further research is needed to advance biomarker identification and side effect mitigation strategies, our findings indicate that patients would likely accept a predictive biomarker for treatment-related toxicities.

## Supplementary Information

Below is the link to the electronic supplementary material.

ESM 1 (DOCX 10.3 MB)

## Data Availability

Privacy restrictions apply to the sharing of University Hospitals Cleveland Medical Center data, which must adhere to University Hospitals’ policies and may require a data use agreement and University Hospital Institutional Review Board approval. Penn State Cancer Institute data may be requested through the corresponding author (C.L.T.).
