# Supplementary material for: Prevalence and predictive testing preferences for breast cancer treatment side effects
Source: Support Care Cancer. 2025 Nov 20;33(12):1095. doi: 10.1007/s00520-025-09976-8 (PMC12630186; doi:10.1007/s00520-025-09976-8)
Supplement: Supplementary file 1 — (DOCX 10.3 MB) [file 520_2025_9976_MOESM1_ESM.docx]

# Supplement

# Supplementary Tables UHCMC Cohort

Supplemental Table 1. UHCMC Cohort Characteristics by Symptoms Experienced

| **Characteristic N(%)**  unless specified | **Symptoms Experienced** | | | |
| --- | --- | --- | --- | --- |
|  | **Endocrine Therapy** | | **Chemotherapy** | Lymphedema (N=130) |
|  | Pain (N=247) | Hot Flashes (N=281) | TIPN (N=86) |  |
| Age, mean (SD) | 60.0 (10.0) | 57.4 (9.8) | 56.4 (8.9) | 59.2 (9.6) |
| Range | 30-83 | 27-82 | 30-73 | 39-81 |
| BMI, mean (SD) | 28.9 (6.4) | 27.8 (6.5) | 29.6 (7.1) | 29.5 (6.7) |
| Range | 19-48 | 17-53 | 18-50 | 18-51 |
| ER+ Tumor | 223 (97.0) | 250 (96.9) | 58 (68.2) | 96 (78.7) |
| Cancer Early-Stage Invasive | 185 (76.8) | 202 (74.8) | 66 (77.6) | 98 (77.2) |
| No Family History of BC | 193 (78.1) | 220 (78.3) | 68 (79.1) | 101 (77.7) |
| Neoadjuvant Chemotherapy | 23 (10.0) | 19 (7.5) | 21 (25.3) | 20 (16.4) |
| Adjuvant Chemotherapy | 82 (36.4) | 84 (33.7) | 56 (70.0) | 55 (45.5) |
| Adjuvant Endocrine Therapy | 206 (94.9) | 231 (95.9) | 52 (65.8) | 87 (75.0) |
| Adjuvant Trastuzumab | 19 (8.2) | 20 (7.7) | 23 (27.4) | 14 (14.8) |
| Breast-Conserving Surgery | 168 (71.2) | 193 (74.2) | 53 (63.1) | 85 (69.1) |
| Radiation Therapy | 151 (68.0) | 166 (67.2) | 63 (75.9) | 81 (69.8) |

*Note: SD=standard deviation; BMI = body mass index; ER+ = estrogen receptor positive; cancer, early-stage invasive = women diagnosed with stage 1 or 2 breast cancer; BC = breast cancer.*

# Supplementary Figures


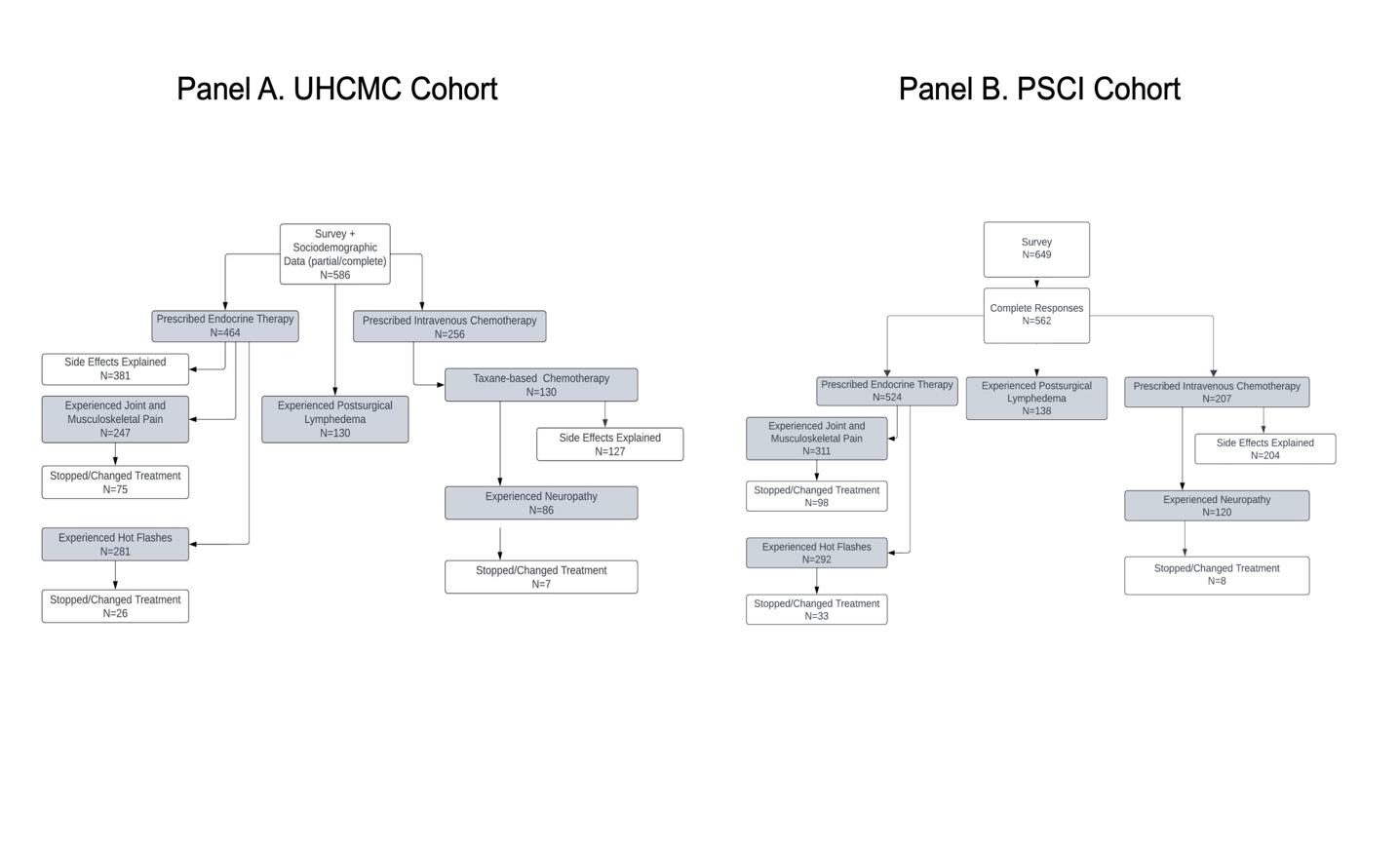


Supplemental Figure 1. Survey flow charts for A. UHCMC Cohort and B. PSCI Cohort

*Note: Treatment and side effect categories are not mutually exclusive, i.e., patients may have received more than one breast cancer treatment and experienced more than one side effect*


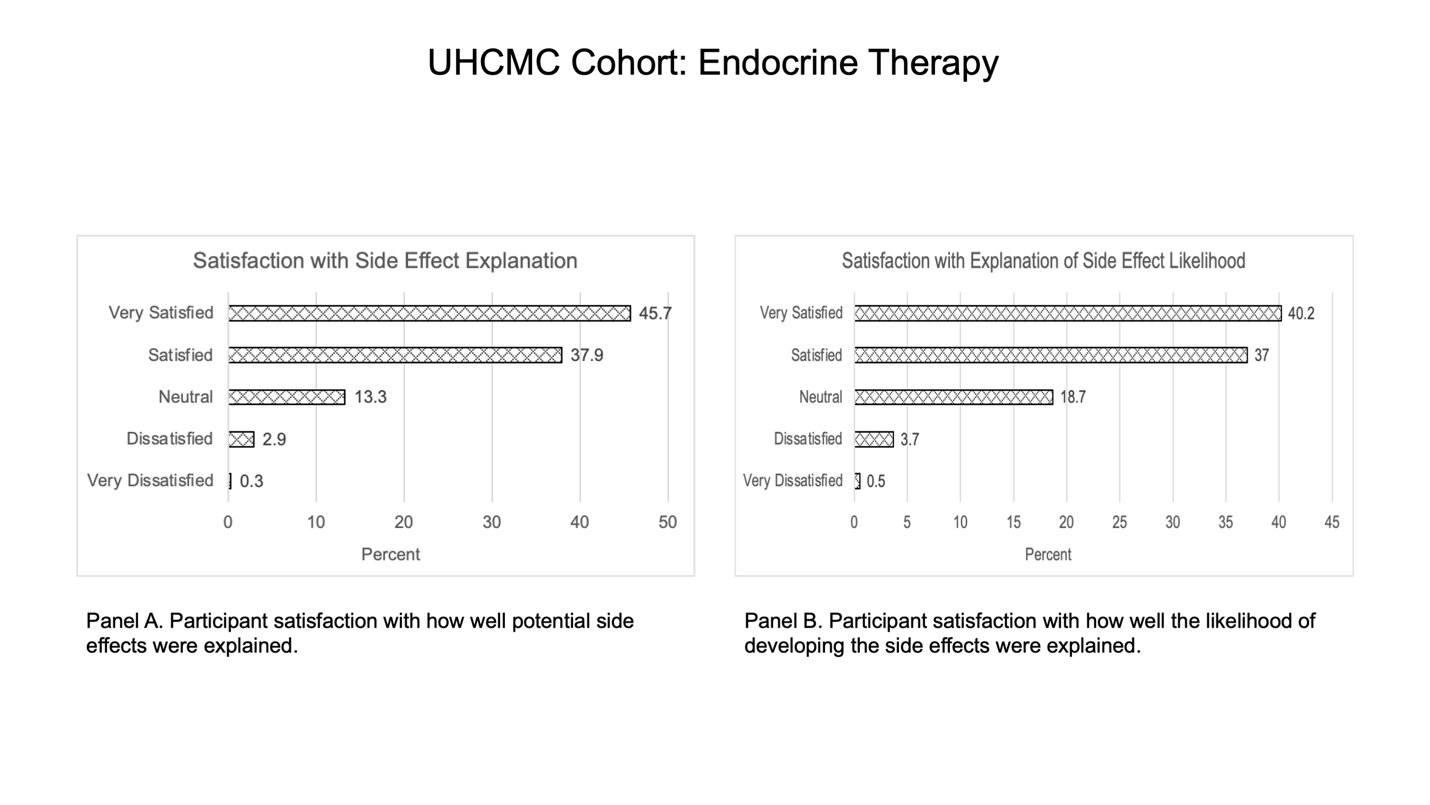


**Supplemental Figure 2**. UHCMC Cohort Endocrine Therapy: Panel A satisfaction with potential side effect explanation with endocrine therapy(N=383); Panel B satisfaction with explanation of likelihood of side effects developing with endocrine therapy (N=378).


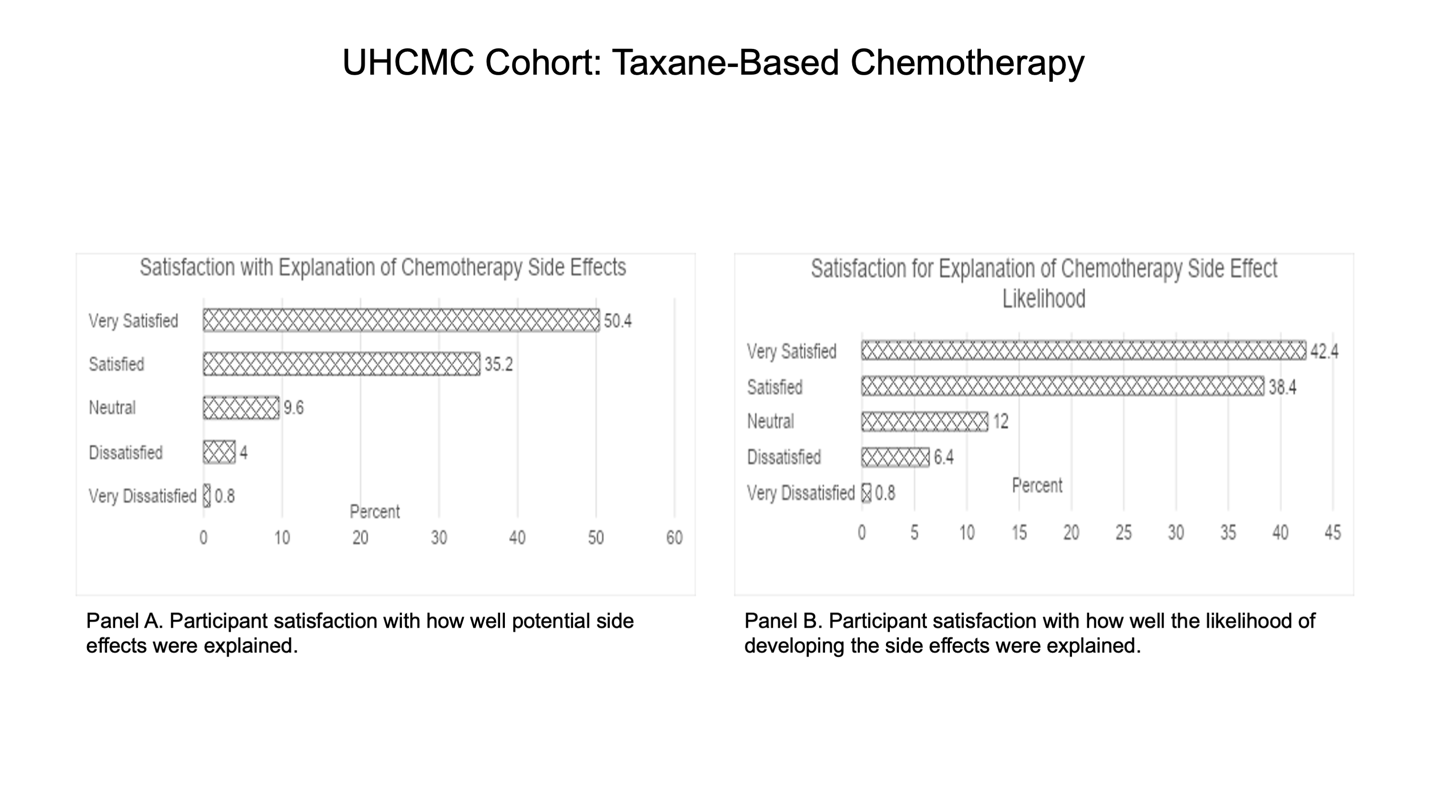


**Supplemental Figure 3.** UHCMC Cohort Taxane-Based Chemotherapy: Panel A satisfaction with potential side effects explanation with taxane-based chemotherapy (N=125); Panel B satisfaction with explanation of likelihood of side effects developing with taxane-based chemotherapy (N=125).

PSCI Cohort: Intravenous Chemotherapy


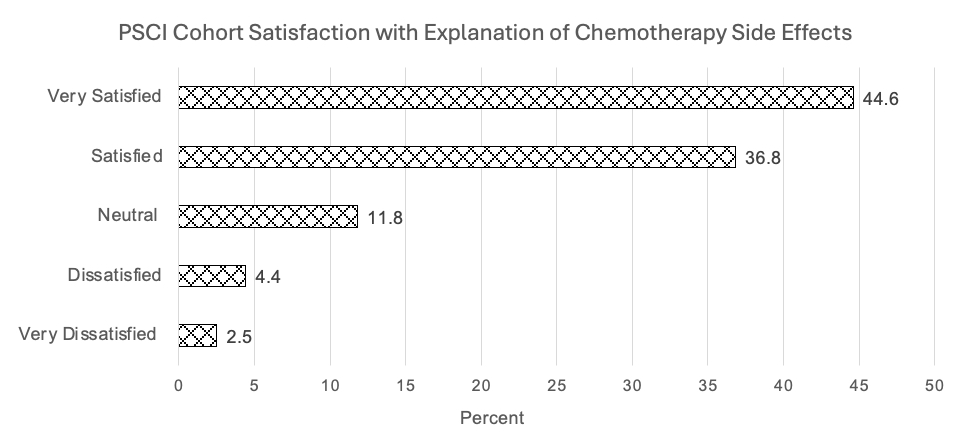


Supplemental Figure 4. PSCI Cohort Satisfaction for Explanation of Intravenous Chemotherapy Likelihood of Developing Side Effects N=204
